# Source‐free domain adaptation for multi‐institutional chest X‐ray images

**DOI:** 10.1002/acm2.70626

**Published:** 2026-05-23

**Authors:** Hyoyi Kim, Seoyoung Lee, Seungryong Cho

**Affiliations:** ^1^ Department of Nuclear and Quantum Engineering KAIST Yuseong‐gu, Daejeon South Korea; ^2^ Department of Radiology Mayo Clinic Rochester Minnesota USA

**Keywords:** ranking‐based pseudo‐labeling, selective fine‐tuning, source‐free domain adaptation, tuberculosis detection

## Abstract

**Background:**

Machine learning models for chest X‐ray (CXR)‐based tuberculosis (TB) detection often suffer from performance drops when deployed across institutions due to domain shifts caused by differences in patient populations, imaging protocols, and equipment. Domain adaptation has been widely studied to address the problem, but privacy regulations restrict data sharing, making conventional approaches infeasible.

**Purpose:**

We aim to enhance TB detection performance when a detector model is shared across multiple clinical sites without accessing any source data or statistics. This problem is related to source‐free domain adaptation (SFDA) but requires special designs for imbalanced binary classification.

**Methods:**

We propose two methods for TB detection, which are based on Source HypOthesis Transfer (SHOT) and SFDA via source Distribution Estimation (SFDA‐DE), respectively. As the main ideas of our methods, we propose ranking‐based pseudo‐labeling and selective fine‐tuning of network layers to address severe class imbalance and prevent overfitting.

**Results:**

On six unseen target domains with high class imbalance, our proposed SFDA methods significantly improved the average F1 score and AUROC compared to non‐adapted models, all without accessing source data during adaptation.

**Conclusions:**

Our methods offer a privacy‐preserving and effective strategy to improve the generalization of TB detection models across institutions, addressing domain shifts while complying with data‐sharing restrictions.

## 1 INTRODUCTION

Machine learning models achieved promising performance for tuberculosis (TB) detection in radiographs. Lakhani and Sundaram^1^ showed that an ensemble of AlexNet and GoogLeNet can perform as well as expert readers in certain conditions, while Hwang et al.^2^ emphasized the importance of lung‐field masking. Subsequent work focused on speed and deployment constraints: Pasa et al.^3^ proposed an efficient MobileNet‐like backbone; Qin et al.^4^ improved robustness with a two‐stage ensemble; and Islam et al.^5^ leveraged weak labels to localize TB lesions. Most recently, Liu et al.^6^ integrated multisite metadata to mitigate demographic bias.

However, these models suffer dramatic performance drops when deployed across institutions due to variability in patient demographics, imaging protocols, and scanner hardware. For example, chest X‐ray (CXR) images collected at different hospitals can differ in bit‐depth, noise characteristics, field‐of‐view, and preprocessing pipelines, introducing a *distribution shift* between source and target domains.^7^, ^8^ Without explicit adaptation, a model trained on one site may fail to generalize, undermining its clinical utility.

Unsupervised domain adaptation has been widely studied to improve model transfer across domains. Representative methods reduce domain discrepancy in feature or image space in various ways.^9^, ^10^, ^11^, ^12^, ^13^, ^14^ However, most methods assume access to source data, source features, or source‐domain statistics during adaptation, which is often impractical in medical imaging because patient data are protected by privacy regulations. Federated learning offers another privacy‐conscious paradigm by training models collaboratively across institutions without exchanging raw images.^15^, ^16^ While effective when all participating sites are available during model development, it is less suited to deployment settings in which a pretrained model must later be transferred to previously unseen hospitals.

Source‐free domain adaptation (SFDA) is therefore an attractive framework for medical imaging deployment because it separates source training from target adaptation and requires only a pretrained source model at the target site^17^, ^18^, ^19^. This setup matches realistic clinical workflows, where hospitals may be willing to share trained models but not patient images or even domain statistics. Despite this, SFDA for medical imaging remains underexplored, especially for disease detection with severe class imbalance. In CXR TB screening, positive cases are rare,^20^, ^21^ making pseudo‐labeling unstable and causing standard SFDA objectives to bias adaptation toward the majority class. In addition, adaptation must remain sensitive to anatomically relevant regions such as the lung fields, since spurious correlations outside the lungs can degrade clinical reliability.^22^, ^23^

In this work, we address these gaps by developing two SFDA methods for TB detection in multi‐institutional CXR data. Building on SHOT^17^ and SFDA‐DE,^18^ we introduce ranking‐based pseudo‐labeling and selective fine‐tuning to improve adaptation under severe class imbalance while reducing overfitting. We first train source models using statistic‐based standardization methods, including histogram normalization and multi‐frequency normalization,^23^ and then adapt them to target domains without access to any source images or source statistics. Through evaluation on six clinical target domains, we show that these designs enable reliable and privacy‐preserving adaptation with substantial gains in predictive performance.

Our contributions are summarized as follows:

(1) **Adaptation strategies tailored for medical challenges**: We introduce ranking‐based pseudo‐labeling and selective fine‐tuning, which are particularly effective in handling severe class imbalance and anatomical relevance–key challenges in clinical CXR interpretation.
(2) **Proposed methods for TB detection**: We propose two novel approaches for TB detection by incorporating our key ideas to SHOT and SFDA‐DE, which are popular algorithms for SFDA, mitigating overfitting that can be observed from the baseline models.
(3) **Improved generalization over standardization techniques**: Our proposed methods significantly enhance performance over baselines, even without access to source data or statistics during adaptation.

## 2 METHODS

We introduce our proposed methods for source‐free domain adaptation (SFDA). We consider Source HypOthesis Transfer (SHOT)^17^ and SFDA via source Distribution Estimation (SFDA‐DE)^18^ as our base algorithms, as they have shown strong empirical performance in previous work regardless of the target domain. We apply ranking‐based pseudo‐labeling and selective fine‐tuning to these algorithms as our key ideas for TB detection.

In addition, we use six different versions of statistic‐based data standardization^23^ in data preprocessing. This is to ensure the consistency with previous work for the TB detection task, although they were proposed for domain adaptation *with source data*, not SFDA.

### 2.1 Problem definition of source‐free domain adaptation

*Unsupervised domain adaptation* aims to adapt a machine learning model trained on a labeled source domain to an unlabeled target domain.^7^, ^24^ Given a labeled source dataset $D_{s}\subset X\times Y$ and an unlabeled target dataset $D_{t}\subset X$, where multiple target domains can be given, we aim to learn a classifier $f_{\theta }:X\mapsto Y$ that performs well in the target domain(s). We assume that the feature space X and label space Y are shared among all domains, for example, their dimensionality, while their marginal distributions may vary.

In *source‐free domain adaptation* (SFDA), the training of a classifier and its test‐time adaptation to the target domains are explicitly separated. Specifically, we cannot access (a) target‐domain data at training time and (b) source‐domain data at test time when we perform actual domain adaptation. SFDA is a more practical scenario than the general domain adaptation; for example, when a hospital distributes a disease‐detection model trained on its patients to other hospitals, the training data of its patients cannot be shared together with the model due to privacy issue.

The evaluation protocol is the same as in typical classification; we compare (unseen) test labels with the model's predictions in the target domains. We assume that there can be severe class imbalances in the target domains, which is common in medical data, especially if the task is to predict the existence of a disease. Therefore, when evaluating the performance on target domains, we perform random under‐sampling in each domain 1,000 times and report the averages and standard deviations. This is done only for evaluation purpose, as the imbalance itself is addressed by our proposed ideas such as creating ranking‐based pseudo‐labels. We use the area under the ROC curve (AUROC) and the F1 score as the main evaluation metrics.

### 2.2 TB detection dataset

Our goal is to design a source‐free domain adaptation method for TB detection given CXR images. We specifically study a multi‐institutional CXR dataset collected from seven clinical sites,^23^ where one of them is the source domain, while the other six are the target domains. Table 1 summarizes their information. The images from different domains are diverse in terms of CXR acquisition settings and bit‐depths, of which details are unavailable. Note that there is class imbalance except for the source domain, ranging from the ratio of 1.8× to 11.4×, making the problem more difficult. Each image is grayscale and rescaled to 512×512.

**TABLE 1:** Dataset statistics. The images are collected from seven different sites. Imbalance is defined as the ratio of normal images over the TB images, ranging from 1.0× to 11.4×.

		# of Images		
Dataset	Use	Normal	TB	Imbalance
Source domain	Training	997	997	1.0×
Source domain	Validation	250	250	1.0×
Target domain A	Test	250	77	3.2×
Target domain B	Test	248	41	6.0×
Target domain C	Test	244	136	1.8×
Target domain D	Test	285	40	7.1×
Target domain E	Test	247	37	6.7×
Target domain F	Test	297	26	11.4×

Each image is paired with a lung mask obtained from a pre‐trained lung segmentation model, which separates an image portion relevant to the target task to improve the efficiency of the main detector. The lung segmentation was conducted in previous work^23^ using a deep neural network having the Res‐UNet structure.^22^ The segmentation network was trained using the JSRT CXR dataset^21^ with its lung mask^25^ as the target. We treat the lung masks as fixed input and will not modify them during the process.

### 2.3 TB detection framework

We use ResNet18^26^ with slight modifications as the detector model. Specifically, we replace the first convolution layer to take gray‐scale images as input, replace the last fully‐connected layer to produce a scalar output for binary classification, and insert a dropout layer^27^ with a dropout probability of 0.6 before the fully‐connected layer. We conduct random parameter initialization in these new layers, while taking the parameters pretrained for ImageNet‐1K^28^ provided by PyTorch^29^ for the other layers.

We chose ResNet18 to provide a lightweight and well‐established baseline for evaluating the proposed source‐free domain adaptation strategy, rather than to claim superiority of a particular architecture. Because the source training set contains only 997 images per class and the target domains are both small and highly imbalanced, using a deeper model such as ResNet50 or DenseNet would increase representational capacity but also introduce additional factors affecting overfitting and adaptation stability.

**FIGURE 1:**
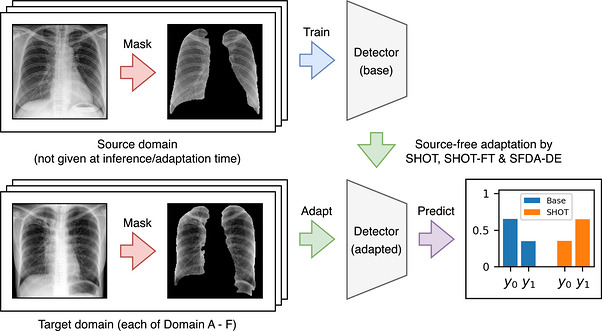
Overview of our source‐free adaptation framework. We train a TB detector using a set of lung‐masked images from the source domain. Then, we adapt the detector with our proposed technique without accessing any of the training images or the target labels. The generated detector makes a better prediction curve compared to the base model.

Since each image is given with its corresponding lung mask, we preprocess the image before giving it to the detection network. Each lung mask is a binary array of size 512×512, which is the same as the image. We multiply the image with its mask, making all pixels outside the lungs as zero, so that the network can focus on the regions of interest. Thus, the network takes a gray‐scale image where a lung mask is already applied. It is noteworthy that different preprocessing methods can be applied depending on the specific algorithm of domain adaptation (see Section 2 for details). Figure 1 illustrates an overview of our TB detection framework with source‐free adaptation.

We use binary cross entropy (BCE) as the objective function for training the network on the source‐domain data. Early stopping is adopted based on the validation loss computed for the same domain. We provide the list of hyperparameters regarding the training of the network in Table 2.

**TABLE 2:** List of hyperparameters for the training of the network.

Name	Value
Maximum number of epochs	1200
Batch size	128
Optimizer	SGD with momentum strength 0.9
Initial learning rate	$10^{-4}$
Weight decay	0.1
Scheduler	Cosine annealing with warm restarts

#### 2.3.1 Notations

We denote the ResNet18 model by f∘g, where f and g refer to the feature extractor (or encoder) and the classifier, respectively, following the convention in previous work. Specifically, g is the last fully‐connected layer including the softmax function, while f includes all convolution blocks. Let $X_{t}$ be the set of images (or image features in general) given at test time. We denote the number of target classes by K, which is in fact fixed as 2 in our TB detection scenario. Let $g{\left(\cdot \right)}_{k}$ refer to the k‐th output of g, where $\sum_{k=1}^{K}g{\left(\cdot \right)}_{k}=1$ for any specific input due to the softmax.

### 2.4 Multi‐frequency‐based data standardization

Various data normalization and standardization methods were used in^23^ to address the distribution shift between source and target domains: (a) normalization, (b) standardization, (c) standardization with lung masking, (d) multi‐frequency‐based standardization, and (e) multi‐frequency‐based standardization with lung masking.

*Normalization (N)* is the simplest approach, which scales the pixel values of target‐domain images to match the minimum and maximum values of the source dataset using histogram clipping (2nd to 98th percentile). While it can effectively adjust image brightness and contrast, it lacks the ability to homogenize texture information.

*Standardization (S) and S with lung masking (SL)* adjust each target image–s mean and standard deviation to align with those of the source domain. The SL method enhances the S method by computing statistical parameters exclusively within the lung regions, identified using the segmentation model. Although this method can improve the consistency of first‐order features (e.g., intensity), it does not address textural variations.

*Multi‐frequency‐based standardization (MFB) and MFB with lung masking (MFBL)* use Laplacian pyramid decomposition^30^ to perform frequency‐specific standardization. Each frequency band of a target‐domain image is adjusted to match corresponding statistical parameters of the source domain. MFBL improves MFB by calculating statistical parameters within the lung regions only, based on the segmentation model.

The application of these methods to a source‐free scenario is limited since the statistics of source data should be available to the target domain. Data privacy is crucial in the medical domain, and sharing data statistics can be considered as exposing an important characteristic of data. For a target‐domain expert (probably in a hospital) who aims to adapt the source model, these techniques are often not available without access to the source statistics.

Therefore, we consider these data standardization methods as the base models to improve upon with our domain adaptation methods, rather than competing approaches. Given six different models trained with different standardization methods, including one without standardization, we aim to improve their performance by applying our approaches for SFDA. In this way, our work can be applied together with those require the statistics or changing the source model for domain adaptation.

### 2.5 SHOT

SHOT operates by transferring a pre‐trained source model instead of using both labeled source data and unlabeled target data for domain adaptation. The method freezes the source model–s classifier and adapts only the feature extractor to the target domain. This adaptation (or fine‐tuning) is guided by an information maximization objective, which encourages confident and diverse predictions on the target data, and thus implicitly aligns target features with the source hypothesis.

To further enhance the feature alignment, SHOT proposes a self‐supervised pseudo‐labeling mechanism. It avoids directly trusting the classifier–s outputs, which can be noisy under domain shift, by computing class‐wise feature prototypes in the target domain and assigning pseudo‐labels based on feature proximity. This strategy refines supervision without any labeled target data and ensures that target representations are better structured for classification under the fixed source hypothesis.

#### 2.5.1 Original formulation

The objective function of SHOT, which is used to fine‐tune the feature extractor while freezing the classifier, consists of three terms:

(1) $$L_{\mathrm{shot}}\left(\theta \right)=L_{\mathrm{ent}}\left(\theta \right)+L_{\mathrm{div}}\left(\theta \right)+\beta \cdot L_{\mathrm{pseudo}}\left(\theta \right),$$

where θ is the set of learnable parameters, β is a balancing hyperparameter between the individual terms, and $L_{\mathrm{ent}}$, $L_{\mathrm{div}}$, and $L_{\mathrm{pseudo}}$ are called the entropy, diversity, and pseudo‐label losses, respectively.

The entropy loss $L_{\mathrm{ent}}$ is defined as

(2) $$L_{\mathrm{ent}}\left(\theta \right)=-E_{x_{t}\in X_{t}}\left[\sum_{k=1}^{K}g{\left(f\left(x_{t}\right)\right)}_{k}\log g{\left(f\left(x_{t}\right)\right)}_{k}\right],$$

As its name implies, this loss measures the entropy of each prediction. Minimizing this loss guides the model to produce a one‐hot‐like prediction for each data, reducing the uncertainty of predictions, working as a self‐supervised objective that boosts the source signals given from the source model.

The diversity loss $L_{\mathrm{div}}$ is defined as

(3) $$L_{\mathrm{div}}\left(\theta \right)=\sum_{k=1}^{K}{\bar{p}}_{k}\log{\bar{p}}_{k},$$

where ${\bar{p}}_{k}=E_{x_{t}\in X_{t}}\left[g{\left(f\left(x_{t}\right)\right)}_{k}\right]$ is the mean prediction on all target data for class k. The diversity loss measures the negative entropy of the mean prediction. By minimizing this term, overall predictions are balanced so that no class takes the vast majority of predictions.

For the pseudo‐label loss $L_{\mathrm{pseudo}}$, we estimate pseudo‐labels of the target samples and then compute the loss term. For estimating the pseudo‐labels, we create the initial centroid of embeddings for each class k as follows:

(4) $$c_{k}^{\left(0\right)}=\frac{\sum_{x_{t}\in X_{t}}g{\left(f\left(x_{t}\right)\right)}_{k}\cdot f\left(x_{t}\right)}{\sum_{x_{t}\in X_{t}}g{\left(f\left(x_{t}\right)\right)}_{k}}.$$

This is the weighted average of target features using their predicted probabilities for class k as the weight. Then, we obtain the initial pseudo‐labels by the nearest neighbor approach:

(5) $${\hat{y}}_{t}^{\left(i\right)}=\underset{k}{arg max}d\left(f\left(x_{t}\right),c_{k}^{\left(i\right)}\right),$$

where d is a similarity function between vectors. i=0 for the initial pseudo‐labels, while i>0 in the subsequent iterations. We use the cosine similarity, that is, $d\left(a,b\right)=a^{⊤}b/∥a∥∥b∥$.

Then, we choose the number of subsequent iterations to refine the pseudo‐labels. Each step consists of the cluster update and the assignment of pseudo‐labels. The cluster update step is given as

(6) $$c_{k}^{\left(t\right)}=\frac{\sum_{x_{t}\in X_{t}}1\left({\hat{y}}_{t}^{\left(i-1\right)}=k\right)\cdot f\left(x_{t}\right)}{\sum_{x_{t}\in X_{t}}1\left({\hat{y}}_{t}^{\left(i-1\right)}=k\right)},$$

where 1(·) returns 1 if the condition is true, and 0 otherwise. This step is averaging the embeddings of target samples weighted by their current pseudo‐labels, and is followed by the assignment step in Equation (5) for higher iteration indices i=1,2,…, chosen as a hyperparameter.

Given the pseudo‐labels after a fixed number of iterations, chosen as a hyperparameter, the pseudo‐label loss is defined as

(7) $$L_{\mathrm{pseudo}}\left(\theta \right)=E_{\left(x_{t},{\hat{y}}_{t}\right)\in X_{t}\times {\hat{Y}}_{t}}\left[-\sum_{k=1}^{K}1\left({\hat{y}}_{t}=k\right)\log g{\left(f\left(x_{t}\right)\right)}_{k}\right].$$

This loss can be understood as measuring the cross entropy between pseudo‐labels and predictions, and is equivalent to using the pseudo‐labels as the answer of predictions.

#### 2.5.2 Our improvement for TB detection

We improve the SHOT framework in three main aspects to make it work better on the TB detection scenario. First, we modify the assignment step of pseudo‐labels in Equation (5) to a ranking‐based approach. Let $s(x_{t})$ be a score vector of $x_{t}$, created by applying the softmax function to the similarity between $f(x_{t})$ and each centroid vector as follows:

(8) $$s{\left(x_{t}\right)}_{k}=\frac{\exp d\left(f\left(x_{t}\right),c_{k}^{\left(i\right)}\right)}{\sum_{k^{\prime }=1}^{K}\exp d\left(f\left(x_{t}\right),c_{k^{\prime }}^{\left(i\right)}\right)}.$$

In our TB detection scenario, k=1 represents the existence of TB, and k=0 represents that the image is normal. Then, the original SHOT framework can be considered as assigning ${\hat{y}}_{t}=1$ if $s{\left(x_{t}\right)}_{1}>\gamma$ independently for each $x_{t}$, where γ=0.5, since $s{\left(x_{t}\right)}_{0}+s{\left(x_{t}\right)}_{1}=1$. However, this approach is highly sensitive to the choice of γ, and setting γ=0.5 does not produce an optimal result due to high class imbalance in our dataset. Accurately tuning γ is itself a challenging problem since we have no target labels.

We propose to rank the scores for all target samples, that is, $\left\{s{\left(x_{t}\right)}_{1}|x\in X_{t}\right\}$, and assign the first τ% to have ${\hat{y}}_{t}=1$. This approach is useful since we can adjust pseudo‐labels not based on the similarity threshold but based on the ratio, which is more interpretable and easier to tune. The value of τ∈(0,1) is a hyperparameter, but we have found from our experiments that simply setting τ=0.5 works well in most cases since the actual predictions are not always aligned with pseudo‐labels; the other loss terms in Equation (1) adjust the actual prediction ratio to be smaller than τ, making a balance between the mode's knowledge and the pre‐defined ratio.

Second, we remove the diversity loss $L_{\mathrm{div}}$ in Equation (3) from the overall objective function. The main reason is because in binary classification, the role of $L_{\mathrm{div}}$ is to make predictions generally balanced between the two classes by regularizing the mean predictions. With our modification of the pseudo‐labeling process, the balance of predictions is guided by the pseudo‐label loss $L_{\mathrm{pseudo}}$ with τ. Thus, the roles of the two loss terms are overlapped, making the model easy to suffer from overfitting.

Third, we modify how to transfer the trained source model f∘g into a target domain. The original framework transfers the classifier g with frozen parameters and updates the feature extractor f in the target domain. This approach is reasonable when there are sufficient target samples, but in a medical domain with limited data, it is easy to cause overfitting and to make the model forget the knowledge of the source domain since f typically contains many parameters. Thus, we freeze all the layers in f except for the last residual block to control the number of parameters to update.

#### 2.5.3 Fine‐tuning with SHOT (SHOT‐FT)

As mentioned previously, a core limitation for applying SHOT to out TB detection scenario is the limited number of target samples. Updating only a subset of layers in f can alleviate the problem, but it also creates unintended bias since the choice of layers to free/update affects the performance. Therefore, we introduce a fine‐tuning approach that adopts the same modified objective function of SHOT, which we call SHOT‐FT. In this approach, we freeze the whole feature extractor f and update the classifier g, changing the *hypothesis* learned for the source domain. In theory, it is hard to expect f to work well on the target domain due to the different distribution of images. However, in practice, we have found that the fine‐tuning version works as well as the version that updates f while freezing g, since fine‐tuning g is much easier than updating f due to less parameters in g.

### 2.6 SFDA‐DE

SFDA‐DE shares a similar motivation with SHOT; it enables source‐free domain adaptation through pseudo‐labels. Without source data at test time, SFDA‐DE estimates the class‐wise source feature distributions by combining target feature statistics with the frozen *class anchors*. This distribution enables the simulation of “source‐like” features for each class.

SFDA‐DE then samples surrogate features from the estimated source distributions and aligns them with the target features using a domain discrepancy loss. This loss function encourages intra‐class compactness and inter‐class separability by minimizing the discrepancy between surrogate source features and target features of the same class. It dynamically updates pseudo‐labels and re‐estimates distributions during training, enabling progressive and effective adaptation. This approach sets new performance benchmarks in the source‐free setting on several domain adaptation datasets.

#### 2.6.1 Original formulation

SFDA‐DE estimates and then uses pseudo‐labels as the main source of information for the objective function. The pseudo‐label of each test sample $x_{t}$ is initialized as

(9) $${\hat{y}}_{t}^{\left(0\right)}=\underset{k}{arg max}w_{k}^{⊤}f\left(x_{t}\right),$$

where $W\in R^{K\times d}$ is the weight matrix in the classifier g trained for the source data, and $w_{k}\in R^{d}$ is the k‐th column of W with d being the embedding size. The vector $w_{k}$ is called the *anchor* for class k and assumed to have knowledge of the source data for class k.

The pseudo‐labels are further updated through iterations via k‐means clustering. The process is the same as the pseudo‐label generation of SHOT, except for the initial centroids created from the anchors as in Equation (9). At each iteration, we compute the new cluster centers from the current pseudo‐labels and then update the pseudo‐labels with the new centers. One difference is that SFDA‐DE has an additional filtering process that discards the pseudo‐labels generated for samples whose distances from the closest centers exceed a certain threshold. However, we do not use this filtering process, as choosing an optimal threshold is not a trivial problem without target labels.

##### 2.6.1.1 Source distribution estimation

In the source distribution estimation step, SFDA‐DE finds the distribution of source data for each class k. Specifically, it assumes the distribution as a multivariate Gaussian $N_{k}\left(\mu_{k},\Sigma_{k}\right)$ and estimates its mean $\mu_{k}$ and covariance $\Sigma_{k}$.

First, we compute a mean embedding for each class k as follows:

(10) $${\bar{h}}_{k}=\frac{\sum_{x_{t}\in X_{t}}1\left({\hat{y}}_{t}=k\right)\cdot f\left(x_{t}\right)}{\sum_{x_{t}\in X_{t}}1\left({\hat{y}}_{t}=k\right)},$$

where 1(·) returns 1 if the condition is true, and 0 otherwise.

Then, the mean vector of the distribution is estimated as follows:

(11) $$\mu_{k}={\left\|{\bar{h}}_{k}\right\|}_{2}\cdot \frac{w_{k}}{∥w_{k}{∥}_{2}},$$

The basic assumption is that the anchor has sufficient information of the source distribution, but its magnitude can change in the target domain due to the distribution shift. Thus, it normalizes the anchor vector and multiplies it with the norm of the class‐conditioned mean of target embeddings.

The covariance matrix $\Sigma_{k}$ is estimated as follows:

(12) $$\Sigma_{k}=\gamma \cdot \frac{H_{k}H_{k}^{⊤}}{\sum_{x_{t}\in X_{t}}1\left({\hat{y}}_{t}=k\right)},$$

where the i‐th column of $H_{k}$ is $f\left(x_{t}\right)-{\bar{h}}_{k}$ where $x_{t}$ is the i‐th sample with ${\hat{y}}_{t}=k$. The value of γ is a hyperparameter and determines the uncertainty of samples in the distribution. This measures the sample covariance of target embeddings conditioned on each class.

##### 2.6.1.2 Objective function

The overall objective function is defined as follows:

(13) $$\begin{array}{c} L_{\mathrm{de}}\left(\theta \right)=\mathrm{MMD}\left(X_{s,0},X_{t,0}\right)+\mathrm{MMD}\left(X_{s,1},X_{t,1}\right)\\ -\mathrm{MMD}\left(X_{s,0},X_{t,1}\right)-\mathrm{MMD}\left(X_{s,1},X_{t,0}\right), \end{array}$$

where MMD represents the maximum mean discrepancy between vector sets, $X_{s,k}$ is a set of artificial source data sampled from the source distribution $N_{k}$, and $X_{t,k}$ is the set of target samples whose pseudo‐labels are k. In general, MMD measures the distance between sets; it becomes zero when the two sets are the same, while increases if they are more different.

Minimizing this objective function induces the (sampled) source data and target data to have similar embeddings for each class k, mainly due to the first two terms. The last two terms make embeddings with different classes distant from each other, creating a more informative feature space. Therefore, this objective function can be understood as a variant of the cross entropy on pseudo‐labels but with more direct manipulation of the feature space not only for the predictions.

#### 2.6.2 Our improvement for TB detection

The application of SFDA‐DE to TB detection is not straightforward, since the classifier g has a single output neuron for binary classification; it can be two based on implementation, but it is considered redundant because if the prediction for a class is p, the prediction for the other class can always be modeled as 1−p with the sigmoid function applied to the predicted logit.

In our setting, the output of g for each sample $x_{t}$ is the score for being TB, and the sigmoid function is applied to make it a probability. This means that we have only a single anchor w, not two for the two classes, and lack the representative information for the normal class. We thus improve SFDA‐DE to have the zero vector as the anchor for the normal class, and set $\mu_{k}$ to the zero vector as well. In addition, we apply the idea of using prior τ to SFDA‐DE for consistency with SHOT. With τ, the assignment of pseudo‐labels is done by sorting all target samples, not independently.

## 3 RESULTS

We present the experimental results of our proposed methods for TB detection, categorized into quantitative and qualitative results. The detector‐training hyperparameters were set to the same values as in previous work to maintain consistency and reproducibility, while the hyperparameters used in the adaptation stage were selected by grid search based on validation data.

### 3.1 Quantitative results

We report the F1 scores and AUROC scores of all variants of our methods in Tables 3 and 4, respectively. Regarding the average F1 scores, all three models significantly improve the baseline performance in almost all combinations of standardization methods and target domains. SHOT‐FT and SHOT improve the average F1 scores by over 20 points and show consistent performance across different target domains. SFDA‐DE also improves the F1 score, but by a smaller amount because its improvement is insignificant in domains C, D, and F, still exhibiting inconsistent performance across domains.

**TABLE 3:** F1 scores with different domain adaptation and data standardization methods.

Adaptation	Data	A	B	C	D	E	F	Average
None	Original	0.411±0.002	0.476±0.006	0.243±0.001	0.182±0.000	0.538±0.016	0.206±0.002	0.343±0.005
	N	0.571±0.007	0.632±0.014	0.386±0.004	0.392±0.007	0.695±0.024	0.264±0.004	0.490±0.010
	S	0.535±0.006	0.553±0.008	0.315±0.002	0.259±0.003	0.596±0.022	0.371±0.006	0.438±0.008
	SL	0.588±0.006	0.619±0.011	0.423±0.004	0.180±0.002	0.687±0.023	0.321±0.004	0.470±0.008
	MFB	0.584±0.004	0.519±0.011	0.286±0.002	0.361±0.006	0.649±0.016	0.264±0.004	0.444±0.007
	MFBL	0.718±0.008	0.570±0.011	0.317±0.002	0.480±0.008	0.598±0.021	0.264±0.005	0.491±0.009
	Average	0.568±0.006	0.561±0.010	0.328±0.002	0.309±0.004	0.627±0.020	0.282±0.004	0.446±0.008
SHOT‐FT	Original	**0.755** ±0.015	**0.742** ±0.024	**0.696** ±0.009	**0.748** ±0.025	**0.734** ±0.024	**0.651** ±0.028	**0.721** ±0.021
	N	**0.757** ±0.015	**0.734** ±0.024	**0.725** ±0.009	**0.757** ±0.025	**0.710** ±0.025	**0.656** ±0.030	**0.723** ±0.021
	S	**0.726** ±0.015	**0.732** ±0.023	**0.713** ±0.009	**0.752** ±0.024	**0.689** ±0.024	**0.697** ±0.031	**0.718** ±0.021
	SL	**0.728** ±0.016	**0.709** ±0.023	**0.677** ±0.009	**0.711** ±0.023	**0.690** ±0.024	**0.707** ±0.029	**0.704** ±0.021
	MFB	**0.739** ±0.016	**0.772** ±0.023	**0.719** ±0.010	**0.698** ±0.023	**0.693** ±0.024	**0.672** ±0.029	**0.716** ±0.021
	MFBL	**0.757** ±0.016	**0.767** ±0.024	**0.688** ±0.009	**0.722** ±0.024	**0.609** ±0.022	**0.739** ±0.031	**0.714** ±0.021
	Average	**0.744** ±0.016	**0.742** ±0.023	**0.703** ±0.009	**0.731** ±0.024	**0.687** ±0.024	**0.687** ±0.030	**0.716** ±0.021
SHOT	Original	**0.753** ±0.016	**0.744** ±0.024	**0.688** ±0.009	**0.722** ±0.024	**0.739** ±0.024	**0.641** ±0.029	**0.715** ±0.021
	N	**0.731** ±0.016	**0.741** ±0.024	**0.715** ±0.008	**0.748** ±0.024	**0.711** ±0.024	**0.640** ±0.028	**0.714** ±0.021
	S	**0.722** ±0.016	**0.724** ±0.024	**0.686** ±0.009	**0.697** ±0.023	**0.670** ±0.023	**0.667** ±0.029	**0.694** ±0.021
	SL	**0.719** ±0.015	**0.727** ±0.023	**0.670** ±0.008	**0.680** ±0.023	**0.696** ±0.025	**0.671** ±0.030	**0.694** ±0.021
	MFB	**0.737** ±0.016	**0.777** ±0.025	**0.676** ±0.009	**0.662** ±0.023	**0.707** ±0.025	**0.691** ±0.030	**0.708** ±0.021
	MFBL	**0.775** ±0.015	**0.753** ±0.023	**0.681** ±0.008	**0.720** ±0.024	**0.624** ±0.022	**0.699** ±0.032	**0.709** ±0.021
	Average	**0.740** ±0.016	**0.744** ±0.024	**0.686** ±0.008	**0.705** ±0.024	**0.691** ±0.024	**0.668** ±0.030	**0.706** ±0.021
SFDA‐DE	Original	**0.487** ±0.003	**0.527** ±0.009	**0.346** ±0.002	**0.365** ±0.004	**0.627** ±0.021	0.206±0.003	**0.426** ±0.007
	N	**0.697** ±0.010	**0.760** ±0.020	**0.525** ±0.005	**0.468** ±0.012	**0.723** ±0.024	0.261±0.006	**0.572** ±0.013
	S	**0.668** ±0.012	**0.696** ±0.013	**0.487** ±0.005	**0.427** ±0.006	**0.663** ±0.023	**0.415** ±0.010	**0.559** ±0.012
	SL	**0.652** ±0.010	**0.712** ±0.019	**0.547** ±0.006	**0.498** ±0.012	0.679±0.022	**0.367** ±0.009	**0.576** ±0.013
	MFB	**0.622** ±0.008	**0.585** ±0.014	**0.375** ±0.003	**0.477** ±0.010	**0.731** ±0.021	0.264±0.005	**0.509** ±0.010
	MFBL	**0.765** ±0.013	**0.665** ±0.016	**0.413** ±0.004	**0.601** ±0.012	**0.648** ±0.024	**0.314** ±0.008	**0.568** ±0.013
	Average	**0.649** ±0.009	**0.657** ±0.015	**0.449** ±0.004	**0.473** ±0.009	**0.678** ±0.023	**0.304** ±0.007	**0.535** ±0.011

*Note*: The bold numbers indicate improved scores compared to those without adaptation.

**TABLE 4:** AUROC scores with different domain adaptation and data standardization methods.

Adaptation	Data	A	B	C	D	E	F	Average
None	Original	0.839±0.014	0.853±0.024	0.765±0.012	0.792±0.028	0.776±0.035	0.719±0.047	0.791±0.027
	N	0.826±0.014	0.836±0.023	0.783±0.012	0.792±0.031	0.751±0.034	0.714±0.040	0.784±0.026
	S	0.801±0.015	0.823±0.022	0.770±0.013	0.800±0.028	0.685±0.037	0.751±0.043	0.772±0.026
	SL	0.786±0.016	0.810±0.022	0.757±0.013	0.774±0.032	0.739±0.032	0.715±0.043	0.764±0.026
	MFB	0.837±0.015	0.852±0.025	0.769±0.012	0.754±0.031	0.776±0.028	0.695±0.048	0.781±0.027
	MFBL	0.847±0.013	0.836±0.024	0.774±0.012	0.765±0.030	0.704±0.034	0.783±0.044	0.785±0.026
	Average	0.823±0.015	0.835±0.023	0.770±0.012	0.779±0.030	0.738±0.033	0.730±0.044	0.779±0.026
SHOT‐FT	Original	**0.848** ±0.014	0.845±0.025	**0.782** ±0.011	**0.812** ±0.030	**0.787** ±0.032	**0.737** ±0.044	**0.802** ±0.026
	N	**0.830** ±0.014	0.832±0.022	**0.795** ±0.012	**0.797** ±0.030	**0.764** ±0.033	**0.724** ±0.039	**0.790** ±0.025
	S	**0.803** ±0.016	0.814±0.022	**0.784** ±0.013	0.798±0.029	**0.700** ±0.037	**0.765** ±0.041	**0.777** ±0.026
	SL	**0.790** ±0.015	0.801±0.022	**0.770** ±0.013	**0.775** ±0.031	**0.741** ±0.032	**0.733** ±0.041	**0.769** ±0.026
	MFB	**0.843** ±0.014	0.851±0.027	**0.792** ±0.012	**0.760** ±0.033	**0.781** ±0.027	**0.704** ±0.044	**0.789** ±0.026
	MFBL	0.841±0.013	0.826±0.026	**0.793** ±0.013	**0.774** ±0.029	**0.706** ±0.033	**0.803** ±0.042	**0.791** ±0.026
	Average	**0.826** ±0.014	0.828±0.024	**0.786** ±0.012	**0.786** ±0.030	**0.747** ±0.032	**0.744** ±0.042	**0.786** ±0.026
SHOT	Original	0.838±0.016	0.847±0.025	**0.783** ±0.012	**0.799** ±0.030	**0.797** ±0.032	**0.731** ±0.046	**0.799** ±0.027
	N	0.820±0.016	0.832±0.024	**0.801** ±0.012	0.788±0.030	**0.765** ±0.031	0.711±0.039	**0.786** ±0.025
	S	0.793±0.016	0.820±0.024	**0.785** ±0.013	0.781±0.025	**0.702** ±0.035	0.741±0.041	0.770±0.026
	SL	0.785±0.017	0.809±0.023	**0.770** ±0.012	0.774±0.032	0.738±0.030	**0.718** ±0.044	**0.766** ±0.026
	MFB	0.833±0.015	0.844±0.027	**0.784** ±0.012	0.748±0.033	**0.789** ±0.026	**0.703** ±0.047	**0.783** ±0.027
	MFBL	0.826±0.015	0.822±0.025	**0.782** ±0.012	**0.773** ±0.029	**0.705** ±0.030	**0.785** ±0.045	0.782±0.026
	Average	0.816±0.015	0.829±0.025	**0.784** ±0.012	0.777±0.030	**0.749** ±0.031	**0.731** ±0.043	**0.781** ±0.026
SFDA‐DE	Original	**0.844** ±0.014	**0.853** ±0.025	0.759±0.011	**0.796** ±0.030	**0.778** ±0.034	**0.728** ±0.047	**0.793** ±0.027
	N	**0.833** ±0.014	0.834±0.023	0.776±0.012	0.785±0.031	0.744±0.036	**0.718** ±0.042	0.782±0.026
	S	**0.805** ±0.015	0.822±0.022	0.756±0.013	0.798±0.027	0.675±0.038	**0.756** ±0.041	0.769±0.026
	SL	**0.788** ±0.016	0.809±0.023	0.748±0.013	**0.775** ±0.032	0.736±0.032	**0.717** ±0.042	0.762±0.026
	MFB	**0.843** ±0.014	0.852±0.026	0.766±0.013	0.752±0.033	**0.776** ±0.029	0.695±0.046	**0.781** ±0.027
	MFBL	**0.853** ±0.014	0.831±0.025	0.774±0.012	0.762±0.030	0.701±0.033	0.779±0.042	0.783±0.026
	Average	**0.828** ±0.015	0.834±0.024	0.763±0.012	0.778±0.030	0.735±0.034	**0.732** ±0.043	0.778±0.026

*Note*: The bold numbers indicate improved scores compared to those without adaptation.

Regarding AUROC scores in Table 4, the improvement is not as significant as for the F1 scores. This is because AUROC is computed by ordering all predictions by their scores, and measures the general quality of ordering rather than binary predictions. Thus, AUROC is generally higher than F1 scores in almost all cases, even for bad models, and it is harder to increase the value even with advanced adaptation techniques. Nevertheless, our SHOT models improve performance, especially when no data standardization method is applied, demonstrating their effectiveness for domain adaptation.

The larger gains in F1 score than in AUROC also have an important clinical interpretation. In TB screening, the practical decision is binary—whether a patient should be flagged for further evaluation—so improvement in F1 indicates that the adapted models achieve a better balance between sensitivity and precision at the operating point used for classification. By contrast, AUROC reflects the overall ranking quality of prediction scores across all thresholds and is therefore less sensitive to corrections of decision bias caused by domain shift and class imbalance.

In our setting, the source‐only model tended to favor the normal class in unseen target domains, especially in more imbalanced sites, whereas the proposed methods shifted the decision boundary to recover more TB‐positive cases. This behavior is clinically meaningful because missing positive TB cases is particularly undesirable in cross‐institutional deployment, and the observed F1 improvement suggests that adaptation improves the usefulness of the model for screening decisions even with modest AUROC changes.

### 3.2 Case studies

**FIGURE 2:**
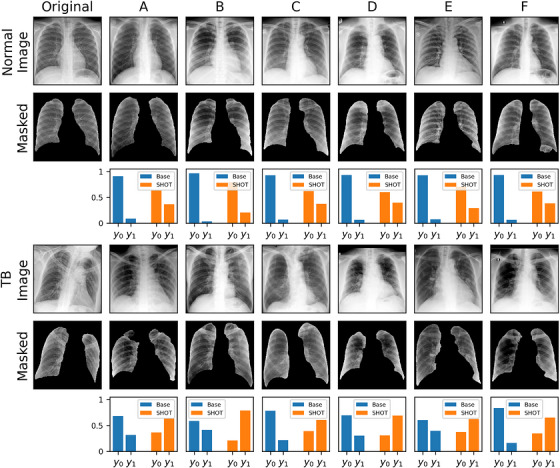
Visualization of masked images from seven different sites, grouped into normal (top) and TB images (bottom), along with predictions from the non‐adapted model (in blue) and our SHOT‐based approach (in orange). The two bars labeled with $y_{0}$ and $y_{1}$ refer to the prediction probabilities for the normal and TB classes, respectively.

We present case studies in Figure 2. For each medical site, we show two masked images, one normal and one TB, along with predictions from the non‐adapted (source‐only) model and from our SHOT‐based model. Images across sites display clear differences; for example, sites C–F include markers of varying shapes and locations, which strengthen domain shift.

The non‐adapted model consistently assigns higher probability to the normal class, failing to adjust to the target sites, whereas our approach correctly predicts the class of every image shown in the figure. Notably, our method exhibits lower confidence for the normal class, which is an expected outcome of adaptation, as it shifts decision boundaries to counter class imbalance by encouraging more abnormal predictions.

### 3.3 Prediction curves

**FIGURE 3:**
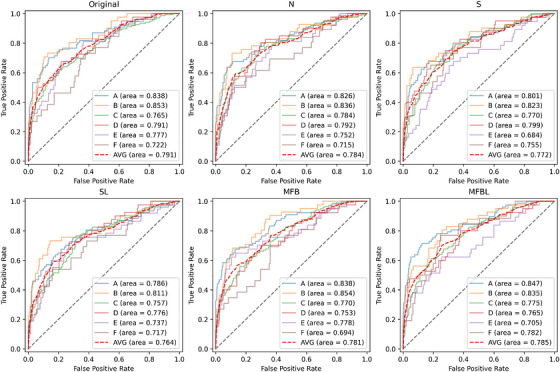
ROC curves with no source‐free domain adaptation method.

**FIGURE 4:**
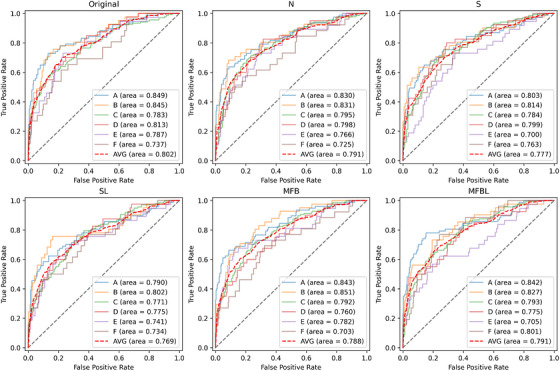
ROC curves when SHOT‐FT is used for domain adaptation.

We visualize the receiver operating characteristic (ROC) curves in Figures 3 and 4 for the baselines and SHOT‐FT, respectively. SHOT‐FT is chosen because it generally shows the best performance among our three methods. The AUROC values in these figures can be different from those in Table 4, since under‐sampling is not performed for the visualization. In accordance with AUROC scores, our SHOT model shows better ROC curves in general, demonstrating its effectiveness, while the difference is small in some cases.

**FIGURE 5:**
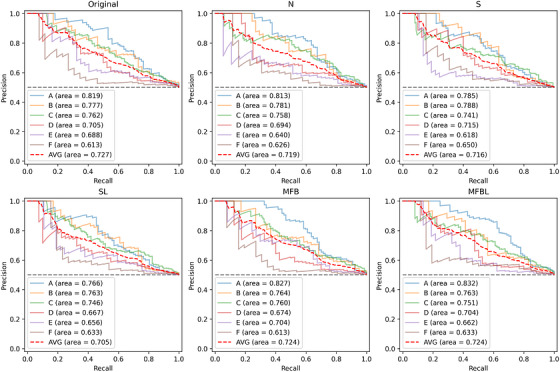
PR curves with no source‐free domain adaptation method.

**FIGURE 6:**
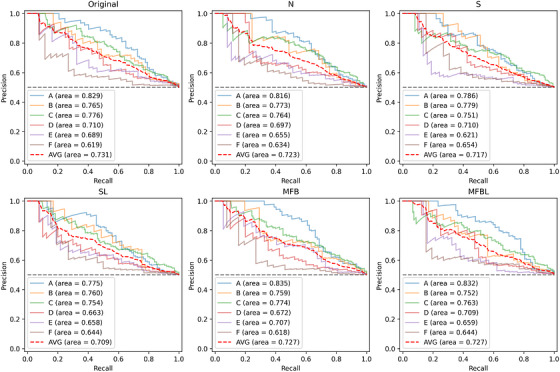
PR curves when SHOT‐FT is used for domain adaptation.

Likewise, we visualize the precision‐recall (PR) curves of the baseline and SHOT‐FT in Figures 5 and 6, respectively. We normalize the precision to have 0.5 as its minimum, since the ratio of TB images is different across target domains and it is hard to compare figures without normalization. SHOT‐FT shows a consistent improvement in AUPRC compared to the original model in all six cases, while the amount of improvement is not as significant as in AUROC due to the different characteristic; AUROC focuses on the general quality of ranking, while AURPC focuses on identification performance.

### 3.4 Adaptation processes

**FIGURE 7:**
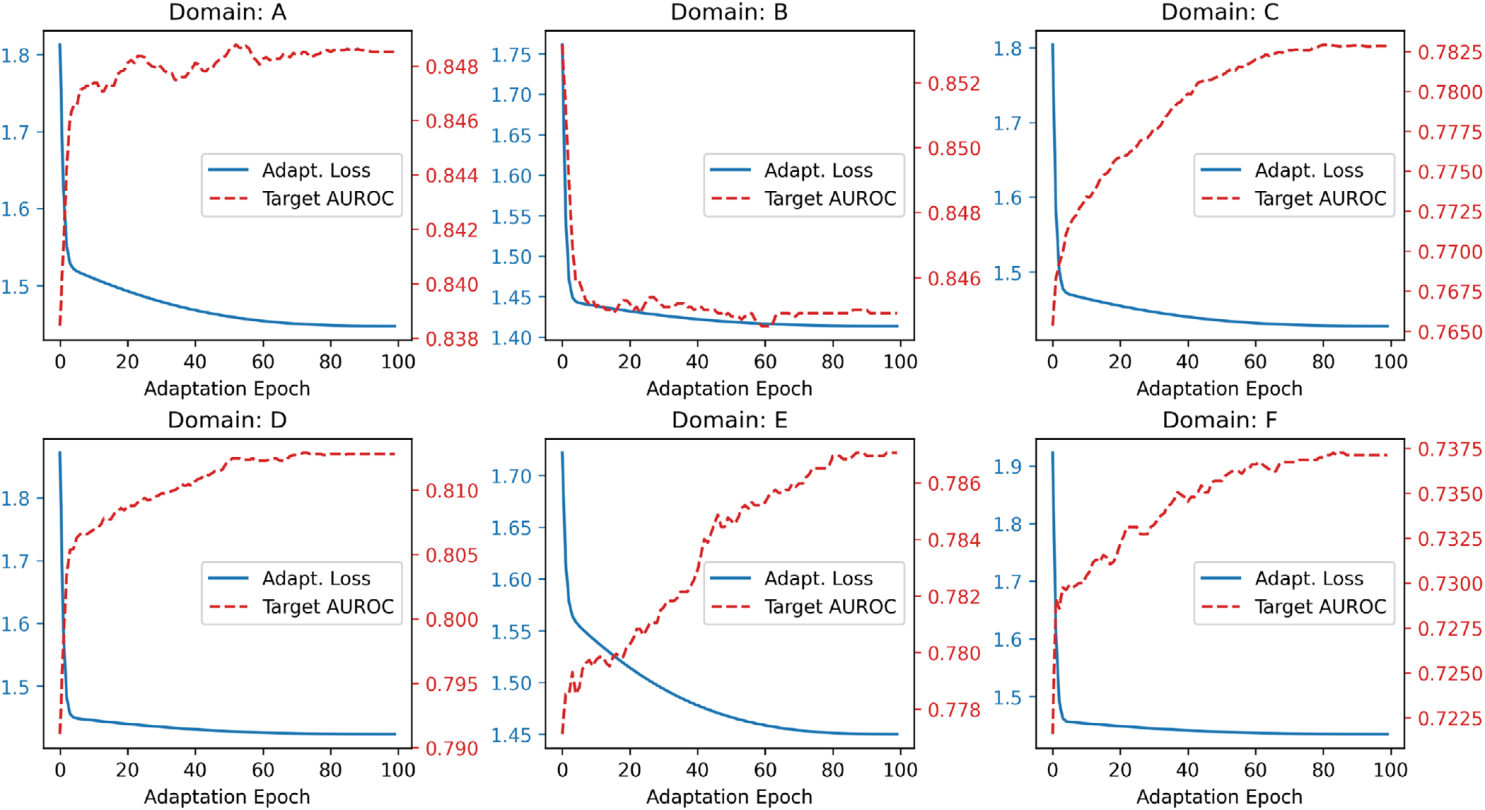
Adaptation losses and target AUROC during the adaptation with SHOT‐FT.

We visualize the change of the adaptation loss (shown in Equation (1)) and the target‐domain AUROC during the adaptation process with SHOT‐FT in Figure 7. The source model does not include any data standardization, while the trend is similar in other standardization methods. In the figure, the loss gradually decreases in all six cases, demonstrating that the model is effectively minimizing the loss. The AUROC increases significantly in five of the six cases, proving its effectiveness, while it fails in one case; our domain adaptation is done completely without test labels, and thus it can fail when the inherent assumption of our adaptation loss is not matched with the true characteristic of the domain.

**FIGURE 8:**
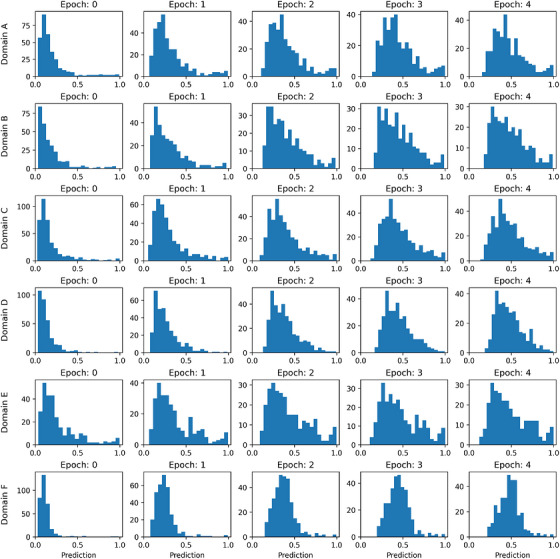
Histograms of TB prediction scores during the adaptation with SHOT‐FT.

We visualize in Figure 8 the histograms of TB prediction scores during the early epochs of domain adaptation with the SHOT framework. In all six target domains, the histograms start with a biased distribution, where almost all prediction scores are below 0.5 due to their domain differences from the source domain used for training the detector. Then, these scores are gradually shifted to the right, thanks to our domain adaptation loss, making a balanced U‐shaped curve, eventually increasing the detection accuracy. The distribution‐level shift is not significant in the later epochs, while the scores are adjusted in a small scale.

### 3.5 Sensitivity study on the value of τ

**FIGURE 9:**
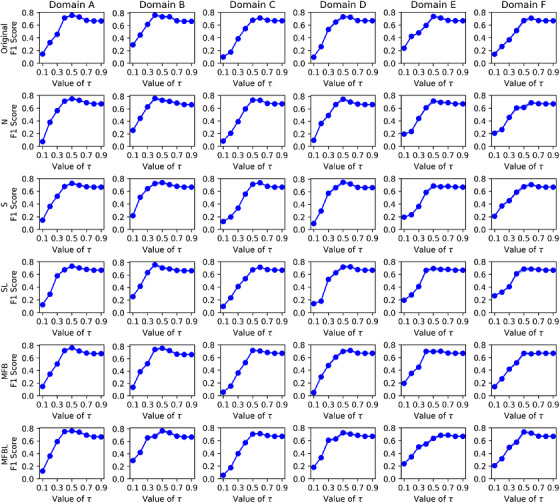
Sensitivity study on the value of τ for SHOT, in terms of the F1 score.

We conducted an ablation study on the prior ratio τ used in SHOT for pseudo‐label assignment. As described in Section 2, τ determines the proportion of target samples that are assigned positive pseudo‐labels after ranking prediction scores. Figure 9 summarizes the F1 scores obtained under different values of τ across all target‐domain transfers. A consistent trend can be observed: when τ is very small, the performance is poor in nearly all cases, while increasing τ rapidly improves the F1 score. The best results are generally achieved in the intermediate range, most often around τ=0.4 to 0.6, after which the performance becomes saturated or slightly decreases.

This behavior is aligned with the role of τ in our framework. If τ is too small, too few target samples are assigned as TB‐positive, leading to overly conservative pseudo‐labels and low recall. In contrast, if τ is too large, the model tends to assign positive pseudo‐labels too aggressively, introducing noisy supervision and reducing precision. The middle range provides a better balance between these two effects, which explains the stable peak of the F1 score. Importantly, τ=0.5 performs competitively in most source‐to‐target settings, supporting our choice of this value as a simple and robust default. Overall, the ablation results indicate that SHOT is not overly sensitive to the exact choice of τ as long as it is selected within a moderate range, which is desirable for source‐free adaptation where target labels are unavailable.

### 3.6 Ablation study on loss terms

**FIGURE 10:**
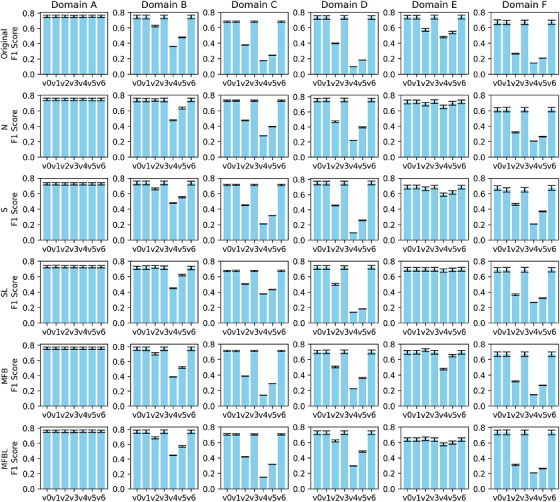
Ablation study on different modules of SHOT, in terms of the F1 score. Refer to Table 5 for more information about the different versions of SHOT.

We further conducted an ablation study to analyze the contribution of each module in SHOT. Figure 10 compares the F1 scores of different SHOT variants across all target domains, while Table 5 summarizes which loss components were included in each version, from v1 to v6.

**TABLE 5:** Different variants of SHOT for an ablation study.

Version	Entropy loss	Diversity loss	Pseudo loss
v0 (Full SHOT)	✓		✓
v1			✓
v2		✓	
v3		✓	✓
v4	✓		
v5	✓	✓	
v6	✓	✓	✓

Overall, the results show that the pseudo‐label loss is the most critical component for achieving strong adaptation performance. Variants that excluded pseudo‐label supervision generally showed substantial performance drops, especially in the more challenging domains such as C, D, and F. This indicates that explicit supervision from pseudo‐labels is essential for guiding the model under domain shift. In contrast, using only entropy or diversity regularization was not sufficient to produce stable adaptation.

Another notable observation is that the diversity loss did not consistently improve performance. In several target domains, versions including the diversity term showed comparable or even worse F1 scores than those without it. This supports our design choice to remove the diversity loss in the proposed SHOT formulation for TB detection. Because our ranking‐based pseudo‐labeling already controls the proportion of positive samples through the prior ratio τ, the diversity term can become redundant and may introduce unnecessary bias, particularly in highly imbalanced binary classification.

The best‐performing variants were those combining entropy minimization with pseudo‐label supervision, and the full combination of all modules did not always yield the highest F1 score. In particular, the ablation results confirm that our modified SHOT benefits mainly from two factors: reliable ranking‐based pseudo‐label assignment and removal of the diversity term, which together provide more stable adaptation across domains.

## 4 DISCUSSION

This work shows that our methods based on source‐free domain adaptation (SFDA) can reliably improve the performance of TB detection models when they are transferred between hospitals that cannot share patient images or even summary statistics. By analysing CXR data from seven institutions whose normal‐to‐TB ratios span 1.8× to 11.4×, we first confirmed how severely a ResNet‐18 detector trained on one site deteriorates on the other six under such distribution shifts.

Building on that, we improved two SFDA algorithms, SHOT and SFDA‐DE, for the TB detection setting. A simple ranking‐based pseudo‐labelling rule produced balanced surrogate labels despite the extreme class imbalance, while selective fine‐tuning of either the last residual block or the classifier head kept over‐fitting in check when only a few target images were available. For SFDA‐DE we devised an alternative anchor for the “normal” class, enabling the method to operate even with only one source logit.

We have a few observations from comprehensive experiments across 36 source‐to‐target transfers. First, the performance improvements of our methods are more significant in F1 scores than in AUROC scores. This is because F1 scores focus on the result of binary prediction, while AUROC scores measure the overall quality of ranked scores. Without explicit target labels, improving the general ranking result is a more difficult problem than tuning the binary decision boundary. Second, the performances of our methods are generally better when based on SHOT than SFDA‐DE. This is because SFDA‐DE relies on the distribution estimation of the source data, which requires complicated estimation and sampling processes, increasing the risk of overfitting without explicit labels.

In particular, the baseline model without domain adaptation shows poor F1 scores in domains C, D, and F. The average F1 score is the lowest in domain F, which has the highest imbalance ratio of 11.4× among the six target domains. Nevertheless, our SHOT‐based methods significantly improve performance in all these domains, achieving the greatest improvement in domain F. This strongly supports the success of our adaptation techniques in extreme cases, where a standard model transfer greatly fails.

Besides the methods explored in our work, alternative strategies include SFDA with information maximization,^31^ adaptive adversarial networks,^32^ contrastive prototype alignment,^33^ statistic‐only model adaptation,^34^ self‐training via domain raptor,^35^ and layer‐wise model surgery.^36^ These works primarily evaluate on natural‐image benchmarks; we extend SFDA to safety‐critical medical imaging where class imbalance and anatomic priors demand additional care in pseudo‐labelling and fine‐tuning.

### 4.1 Limitations

A potential limitation of this study is that the lung masks were generated by a fixed segmentation model trained on the JSRT dataset and were applied to all target domains without further adaptation. Because pixel‐level lung annotations were not available for the external target datasets, the segmentation performance of this model could not be directly evaluated on those domains using metrics such as Dice score or IoU. As a result, we cannot exclude the possibility that domain shift affected the quality of the generated masks, which may in turn have influenced the downstream TB detector.

We chose not to adapt the mask generator in order to keep the scope of the study focused on source‐free adaptation of the TB classification model itself. Introducing segmentation adaptation would add another source of variation and make it more difficult to isolate the contribution of the proposed adaptation strategy. In addition, under the source‐free setting considered here, neither source data nor target segmentation labels were available, making principled adaptation of the segmentation model nontrivial. Future work should investigate joint source‐free adaptation of lung segmentation and disease classification to further improve robustness across datasets.

A further limitation of this study is that the TB detector was built on ResNet18, which is relatively simple compared with deeper architectures such as ResNet50, DenseNet, or more recent medical imaging models. Although this choice allowed us to use a lightweight and stable baseline and to focus on the effect of the proposed source‐free adaptation strategy, it may have limited the representational capacity of the model for capturing more subtle TB‐related patterns. Therefore, the reported performance should be interpreted as validation of the adaptation framework on a standard backbone rather than as the best achievable performance for the task. Future work should examine whether the proposed method can be further improved and generalized when combined with deeper or domain‐specific architectures.

### 4.2 Comparison with recent work

Recent medical‐imaging studies provide an important context for our work. In CXR analysis, several domain adaptation methods have been proposed to improve robustness across institutions, but many of them still require access to source‐domain images or multi‐source training. For example, SPA‐UDA^37^ preserves disease semantics through adversarial image translation for CXR disease recognition, WDDM^38^ aligns CXR domains using Wasserstein distance and discrepancy‐based objectives, and DELCOM^39^ improves unseen‐domain thoracic disease classification through cross‐domain mixup and domain‐ensemble learning. While these studies are highly relevant to clinical deployment, they do not address the strict source‐free setting considered here.

Closer to our formulation, Qiu proposed a causality‐inspired source‐free domain adaptation framework for medical image classification, including experiments on pulmonary CXR abnormalities, by reducing confounding from irrelevant background and source‐model bias through causal interventions.^40^ More recently, Ma et al. introduced a source‐free semi‐supervised domain adaptation method for TB recognition based on a bilateral‐branch consistency network.^41^ In contrast, we focus on fully unsupervised source‐free adaptation for multi‐institutional TB detection under severe class imbalance. In particular, our ranking‐based pseudo‐labeling explicitly controls the positive pseudo‐label ratio, and our selective fine‐tuning strategy reduces overfitting when target‐domain data are limited. These task‐specific designs are important in TB screening, where positive cases are rare and naive pseudo‐labeling can easily collapse toward the majority class.

## 5 CONCLUSION

Our work proposes two novel approaches for TB detection based on source‐free domain adaptation (SFDA). Comprehensive experiments across 36 source‐to‐target transfers confirmed the value of our key ideas: we raised the mean F1 score from 0.447 to 0.716 and AUROC from 0.782 to 0.793 without revisiting the source data. The greatest gains appeared when no handcrafted data standardization was applied, suggesting that our ideas can replace, rather than augment, statistics‐sharing pipelines. Although a few domain pairs still exhibited minor drops, each target site benefited from at least one of our variants, underscoring the need to maintain multiple adaptation strategies in practice.

Our work also reveals the limitations and challenges of SFDA for medical imaging. We focused on a single pathology and binary classification, while more diverse thoracic diseases may demand richer anchors or class‐specific priors depending on the adaptation method. We occasionally observed adaptation failures when the SHOT loss decreased but AUROC worsened, indicating that optimization objectives do not always align with clinical utility. Finally, all evaluations were retrospective on static test sets; prospective studies are needed to confirm robustness as acquisition pipelines evolve.

Future work should therefore focus on adaptive loss scheduling to halt harmful fine‐tuning, semi‐supervised extensions that exploit a handful of on‐site labels, and further validation on modalities such as CT, MRI or ultrasound, as well as on multi‐label and segmentation tasks. By addressing the twin challenges of privacy preservation and domain shift, we chart a realistic path toward equitable, safe deployment of AI‐assisted TB screening tools.

## AUTHOR CONTRIBUTIONS

Hyoyi Kim designed the source‐free domain adaptation framework, conducted the experiments, and drafted the manuscript. Seoyoung Lee reviewed the manuscript and contributed to its revision. Seungryong Cho supervised the study and contributed to manuscript revision.

## CONFLICT OF INTEREST STATEMENT

The authors declare no conflicts of interest.

## ETHICS STATEMENT

This study did not involve any direct experimentation on human subjects, the collection of new human data, or the use of personally identifiable information. All analyses were conducted on de‐identified, retrospective datasets that were made available for research purposes. As such, ethical approval and informed consent were not required under current institutional and national guidelines. The research complies with all relevant laws, ethical standards, and institutional policies regarding the use of anonymized medical data.

## Data Availability

The multi‐institutional chest X‐ray data analyzed in this study were obtained from the dataset described in Kim et al.^23^ and are subject to institutional and patient‐privacy restrictions. Access to the data may be requested from the original data providers under their respective data‐use agreements. The trained source models and adaptation code that support the findings of this study are available from the corresponding author upon reasonable request.
